# Case Report: Two cases of hereditary angioedema in a Chinese family

**DOI:** 10.3389/falgy.2025.1587904

**Published:** 2025-06-02

**Authors:** Yuanli Guo, Manli Qi, Jinluan Ding

**Affiliations:** Department of Dermatology, Tianjin Union Medical Center, The First Affiliated Hospital of Nankai University, Tianjin, China

**Keywords:** family survey, C1-inhibitor, case report, hereditary angioedema (HAE), bradykinin

## Abstract

**Background:**

Hereditary angioedema (HAE) is a life-threatening condition characterized by repeated asymmetric cutaneous and mucosal edema. It is a rare autosomal dominant genetic disease with a mortality rate of 8.6%. Family survey of HAE in China is seldom reported since it is still under recognized.

**Case report:**

We reported two cases of HAE and a family survey conducted in Hebei Province, China. The proband was a woman who had edema for over 7 years. She was diagnosed with type I HAE in her 50s after a life-threatening asphyxia attack. Her elder brother was initially diagnosed with mild symptoms.

**Conclusion:**

Two diagnosed and three suspected patients were identified in our family survey. Family surveys are important method for identifying asymptomatic patients and preventing attacks. It is valuable for rescuing people from sudden death, particularly from asphyxia.

## Introduction

Hereditary angioedema (HAE) is a rare, autosomal dominant, genetic disease. According to a worldwide epidemiological investigation, it has a morbidity rate of 0.13–1.6 in 100,000 (1). In China, the prevalence of HAE in patients with decreased complement 4 levels has been reported to be 2.43 cases per 10,000 (2). One of nine studies that reported the lifespan of patients with undiagnosed HAE type 1/2 who died of asphyxiation was shorter than that of patients with undiagnosed HAE type 1/2 who died of other causes (40.8 years vs. 72.0 years) (3). Therefore, early recognition and diagnosis are important for families with HAE to identify asymptomatic patients before the condition aggravates. Family surveys are the most commonly used method as recommended by the World Allergy Organization (4). Notably, a family survey that has been conducted in China since 1980 found over 400 patients from 120 families with HAE, which implies significant underdiagnosis nationally. The median age of onset in Chinese patients was in the teens (50.5%) and twenties (31.8%) (5). Herein, we report two cases of HAE and a family survey conducted in Hebei Province, China. The proband had a first attack at 54 years of age, which was later than.typical for HAE. All participants provided written informed consent for blood testing and the publication of their clinical information and test results. This study was approved by the Institutional Review Board of the Tianjin Union Medical Center.

## Case report

**Figure 1:**
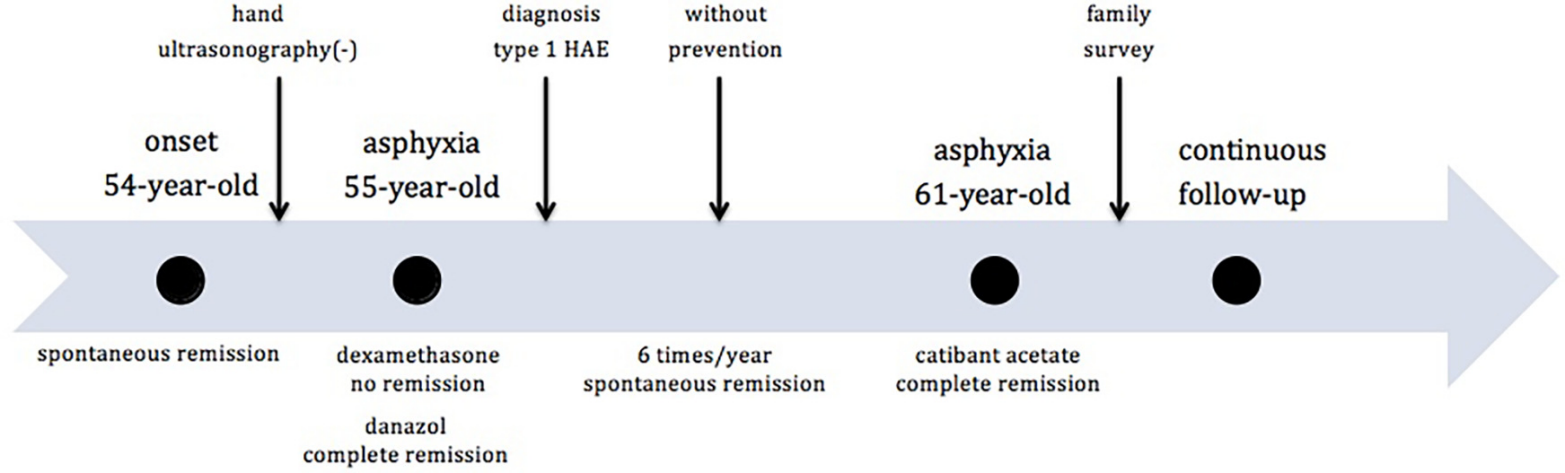
Timeline of proband's episodes.

A 61-year-old woman (proband, ZHY) initially experienced angioedema during her 54th year of life. The patient had no significant history of illness. She experienced angioedema approximately six times per year, with each episode resolving spontaneously within five days. The sites of edema included the hands, feet, cheeks, lips, eyelids, neck, and legs. The most common sites were the hands and feet. The attacks were consistently triggered by physical factors (pressure, minor injury, heavy lifting, or prolonged sitting) and were not accompanied by itching or pain. Hand ultrasonography did not indicate any disease at the time of edema. Her father and older brother had similar symptoms of edema. She had experienced neck edema and asphyxia once in her 55th year of life. Dexamethasone was administered in the emergency department; however, the symptoms remained unresolved. The patient achieved a complete remission after danazol treatment. Due to her repeated edema and positive family history, emergency physicians prescribed laboratory tests for diagnosis. The investigations showed extremely low levels of C1-INH and C4 (Table 1). Quality of Life (QOL) score was seven points out of 36 (Table 2). Hereditary angioedema of type I was diagnosed at this time based on markedly reduced C1-INH level and function (confirmed twice) with low C4 according to its guidelines (4). The differential diagnosis was acquired angioedema with C1 inhibitor deficiency (AAE-C1-INH) due to the relatively late onset of symptoms. AAE-C1-INH showed symptoms similar to those of HAE, characterized by a later-onset age of over 40 years, negative family history, and lower C1q levels. A positive family history and normal C1q level (Table 1) further confirmed the diagnosis. In the years following the diagnosis, angioedema recurred and was relieved without any preventive treatment. One case of life-threatening edema was treated with a 30 mg icatibant acetate injection in her 61st year and was relieved within two hours. The timeline of the key events was shown in Figure 1. The patient underwent continuous follow-up.

**Table 1:** Experimental results of two diagnosed patients. Dried blood spot sample was tested using MS. Serum sample was tested using NMA, IMA, and ELISA.

Name	Item	Value			
		MS	NMA	IMA	ELISA
ZHY (No.2)	fC1-INH	<7.0 (Ref: ≥ 58.9%)			0.0 (Ref: ≥ 68%)
	C1-INH	19.79 (Ref:81.46–291.29*μ*g/ml)	0.03 (Ref:0.21–0.39 g/L)		
	C4	24.92 (Ref:72.85–372.95μg/ml)		0.01 (Ref: 0.1–0.4 g/L)	
	C1q			7.3 （Ref: 5.0–8.6 mg/dl）	
ZYM (No.1)	fC1-INH	<7.0 (Ref: ≥ 58.9%)			0.0 (Ref: ≥ 68%)
	C1-INH	24.20 (Ref:81.46–291.29 μg/ml)	0.04 (Ref:0.21–0.39 g/L)		
	C4	44.72 (Ref:72.85–372.95 μg/ml)		0.06 (Ref: 0.1–0.4 g/L)	

MS, mass spectrometry; NMA, nephelometric assay; IMA, immunoturbidimetry assay; ELISA, enzyme-linked immunosorbent assay; Ref, reference range; C1-INH, C1 esterase inhibitor; fC1-INH, C1 esterase inhibitor function.

**Table 2:** QOL scale used to evaluate the degree of influence that hereditary angioedema has on a patient's daily life. A total of 12 questions were asked. A score of up to 36 points was calculated based on the score of each answer (0-never, 1-light, 2-moderate and 3-severe).

Questions	0, Never	1, Light	2, Moderate	3, Severe
Does your skin feel uncomfortable?				
Do you feel sad, embarrassed, or depressed because of your skin problems?				
Do your skin problems prevent you from shopping or housework?				
Do you choose special clothes or shoes because of your skin problems?				
Do your skin problems prevent you from social communication, outings, or entertainment?				
Do your skin problems prevent you from sports?				
Do your skin problems prevent you from work or study?				
Do your skin problems have any influence on the relationship between you and your spouse, friends, or relatives?				
Do your skin problems bring inconvenience in your daily life?				
Do your skin problems prevent you from sleep?				
Do your skin problems occupy a lot of your time or attention?				
Do your skin problems become an economic burden?				

QOL, quality of life, HAE, hereditary angioedema.

## Family survey

**Figure 2:**
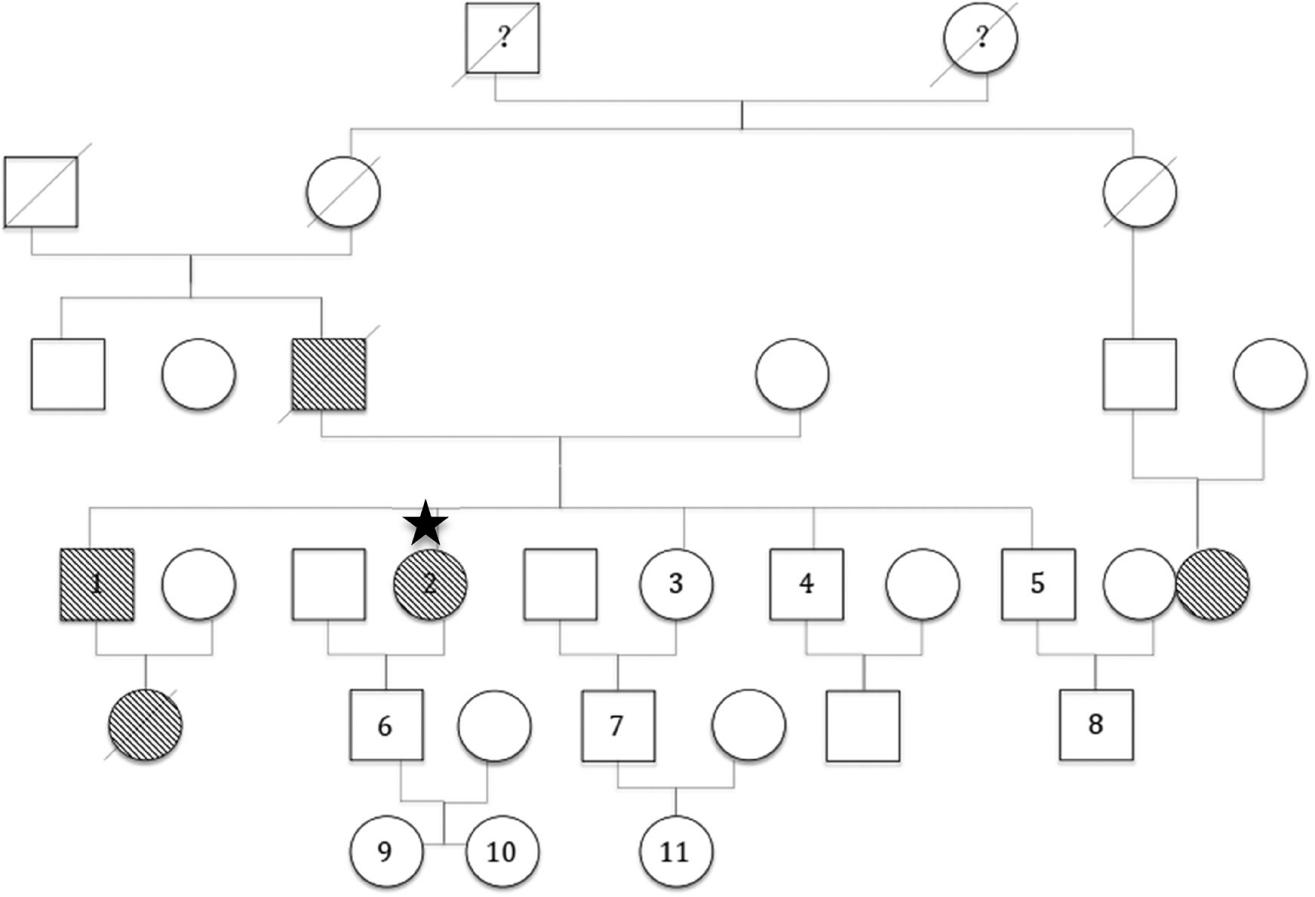
Family tree. 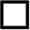: male ○: female: 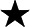 proband. 1–11: Subjects No.1 to No.11 in the proband's family. 1: ZYM. 2: ZHY. ?: It is unknown whether the patient had angioedema symptoms 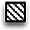 or 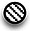: male or female, respective, who had angioedema symptoms. 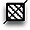 or 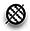: male or female, respectively, who died of suspected hereditary angioedema. 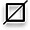 or 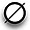: male or female, respectively, who died of another disease.

Family investigations were initiated immediately after the patient visited the outpatient department for treatment. Ten family members from three generations were investigated, including their relationships with the proband, medical history, detailed angioedema history, and treatment. C1-INH, fC1-INH (a functional C1 inhibitor), and C4 were tested using two different methods to avoid errors. QOL was designed related to HAE Three generations of information from parents, grandparents, and great-grandparents were provided according to the memory of the proband. Only one member (NO.1) had edema symptoms, whereas the others were asymptomatic. The family tree was shown in Figure 2.

Four individuals from three generations exhibited angioedema symptoms, excluding the proband. Two of them [the father and the niece (brother's daughter) of the proband] died of asphyxia without an HAE diagnosis. A second cousin also had angioedema symptoms; however, the details were unclear because of a lack of close connection. The proband's elder brother (ZYM) had extremely low C1-INH, fC1-INH, and C4 levels similar to those of his sister (Table 1). The patient initially experienced angioedema in the 22nd year, which resolved spontaneously. Symptoms occurred twice a year and were relieved within five days. The patient had never experienced any life-threatening angioedema. The most common sites were the hands, feet, upper limbs, and genitals. The main causative factor was heavy weight lifting without any accompanying symptoms. He did not recognize it as a disease; therefore, he never visited the hospital for diagnosis or treatment. A score of zero indicated no QOL impact. All other living tested relatives were found to have normal C1-INH, fC1-INH and C4 levels.

## Discussion

HAE is a life-threatening disease characterized by repeated asymmetric cutaneous and mucosal edema. It also causes extreme abdominal pain, accompanied by nausea and vomiting. The estimated risk of death due to asphyxiation is 8.6% (1). Patients initially visit the dermatology department, allergy department, or emergency room. HAE is often unrecognized and misdiagnosed in clinical settings because of low awareness. Depression, anxiety, stress, and alexithymia are the most common emotional symptoms.

HAE is grouped into three types based on the C1-INH levels. Patients with type I subtype have low levels of C4, C1-INH, and fC1-INH. Those with type II subtype have low levels of C4 and fC1-INH but normal levels of C1-INH. Patients with HAE-nC1-INH subtype have normal levels of C4, C1-INH, and fC1-INH. Types I and II are caused by a functional C1 inhibitor deficiency, which leads to an increase in bradykinin-mediated vascular permeability (6). Mutations in SERPING1 may be the mechanism underlying these two types. The transmission pattern of the SERPING1 variant favors the transmission of wild-type alleles in males, especially when the father is a carrier (7). This presents a significant challenge in the diagnosis of HAE-nC1-INH. Family history, plasminogen (8) levels, factor XII (9), kininogen-1, myoferlin, heparan sulfate-glucosamine 3-sulfotransferase 6, and angiopoietin-1 (10) gene missense variants may help in the diagnosis. The World Allergy Organization (WAO)/European Academy of Allergy and Clinical Immunology (EAACI) guidelines for HAE recommend testing fC1-INH, C1-INH, and C4 levels twice for diagnosis, and also testing for known mutations of HAE-nC1-INH (4).

Early on-demand treatment has also been considered. Patients should be well-educated in HAE and trained in self-treatment. The first-line treatment is an intravenous plasma-derived C1 inhibitor (11)(Berinert, Cinryze, or Ruconest), a kallikrein inhibitor (12) (ecallantide), or a bradykinin B2 receptor antagonist (13) (icatibant). When these therapies are not available, SDP (solvent detergent-treated plasma) or FFP (fresh frozen plasma) is used as an alternative treatment (14). If the edema progresses in the airways, intubation or surgical airway intervention should be considered. Prophylaxis is an important component in disease management. Short-term prophylactic treatments, such as intravenous pdC1-INH (plasma-derived C1 inhibitor) or FFP, should be administered before medical, surgical, or dental procedures (15). Long-term prophylactic treatment is used to improve patients' quality of life by completely controlling HAE. The recommended medicines are pdC1-IN (16), lanadelumab (17) (anti-active plasma kallikrein monoclonal antibody), berotralstat (18) (plasma kallikrein inhibitor), androgens (19), and antifibrinolytics (20). The CRISPR/Cas9 gene editing technology may be an effective method for further treatment (21).

In our patient, the proband was not initially on prophylaxis after diagnosis according to the guideline recommendation. At that time (in 2017), prevention and treatment resources of HAE were extremely insufficient in China. Available treatments were limited to freshly frozen plasma or danazol. First-line therapies were not always available in all hospitals and are not known by all emergency physicians. Short-term prophylaxis was initiated five days before triggering with danazol or tranexamic acid. Long-term prophylaxia was rarely applied because of side effects. With the continuous development of new drugs, she was injected with icatibant acetate for a life-threatening attack that occurred at 61 years of age, which provided complete relief. She was intended to receive lanadelumab for a long-term prophylaxia. Notably, a recent survey identified the experiences of long-term lanadelumab prophylaxis in China (22), which led to a 97.8% reduction in the attack rate. Because her brother had mild symptoms and no significant life burden, he refused any preventive methods. We advised him to avoid relevant triggers and emphasized on emergency hospitals. They all prepared one acute attack medication at home.

Family surveys of HAE in China have seldom been reported because it is still under recognized. The most important finding in our case was the late-onset disease in the proband. Although most patients with HAE show symptoms in young adulthood, occasional cases of later manifestations have been reported. The family's story highlights that the absence of symptoms in youth does not preclude HAE, especially if a parent is affected.

## Conclusion

Herein, we reported a case of family screening for HAE in China. Family screening is important to identify patients with mild of no symptoms. It is valuable on saving lives by identifying hidden cases. In this case study, screening of the family of the initial patient revealed a second patient diagnosed with type I HAE and three suspected patients. Experts who focus on rare diseases may present the clinical manifestations and experimental information disclosed in this report in Chinese.

## Limitations

Unfortunately, two of the suspected patients died; therefore, we could not perform any diagnostic tests. Further investigations could be carried out if the proband had a close familial connection with a third suspected patient with angioedema symptoms. We attempted to persuade the two diagnosed patients to undergo further gene detection for HAE; however, they refused. If genetic data were available, it would provide a better understanding of the mutations-identity in the suspected patients.

## Data Availability

The original contributions presented in the study are included in the article/Supplementary Material, further inquiries can be directed to the corresponding author.
